# Does playing video games effect cognitive abilities in Pakistani children?

**DOI:** 10.12669/pjms.346.15532

**Published:** 2018

**Authors:** Aliya Hisam, Syed Fawad Mashhadi, Mahum Faheem, Mahrukh Sohail, Bilal Ikhlaq, Irfan Iqbal

**Affiliations:** 1 *Dr. Aliya Hisam, MBBS, MPH, FCPS (Community Medicine). Associate Professor, Department of Community Medicine, Army Medical College, National University of Medical Sciences, Rawalpindi, Pakistan*; 2 *Dr. Syed Fawad Mashhadi, MBBS, MPH, MPhil. Assistant Professor, Department of Community Medicine, Army Medical College, National University of Medical Sciences, Rawalpindi, Pakistan*; 3 *Dr. Mahum Faheem, MBBS. House Officers, Combined Military Hospital, Rawalpindi, Pakistan*; 4 *Dr. Mahrukh Sohail, MBBS. House Officers, Combined Military Hospital, Rawalpindi, Pakistan*; 5 *Dr. Bilal Ikhlaq, MBBS. House Officers, Combined Military Hospital, Rawalpindi, Pakistan*; 6 *Irfan Iqbal, MBBS. House Officers, Combined Military Hospital, Rawalpindi, Pakistan*

**Keywords:** Cognitive abilities, Gamers, Knowledge, Intelligence, Non-gamers, Teenagers, Video games

## Abstract

**Objective:**

To find out the effect of playing video games on cognitive abilities among teenagers.

**Methods:**

A comparative cross sectional study was conducted at two schools in Rawalpindi from August 2014 till February 2015. A sample size of 171 was calculated by using WHO sample size calculator using non-probability convenient sampling technique. A mixed questionnaire was used as a data collection tool and two groups were defined as video gamers and non-video gamers. Video gamers were those students who were using video games for two hours or more. Cognitive ability of the students were assess by standardized questionnaire named Wonderlic Cognitive Ability Test Questionnaire. Data were entered and analyzed in SPSS version 20.

**Results:**

There were 93 (54.4%) gamers whereas 78(45.4%) were non-gamers. Mean age of the participants was 18.86+1.46 years. Gamers with correct answers to knowledge, analogy, processing speed, deductive reasoning, mathematical intelligence were 57(61.3%), 67(72%), 68 (73%), 58 (62.4%) and 73(78.5%) respectively. Significant association was found between gamer status and gender (p=0.023), analogy (p=0.049), processing speed (p<0.001), deductive reasoning (p=0.003) and mathematical intelligence (p<0.001). There was no significant association of gamer status with knowledge (p=0.188).

**Conclusion:**

Gamers exhibit better range of cognitive abilities specifically involving analogy, processing speed, deductive reasoning and mathematical intelligence. In this study, those who play video games on long term basis, showed improvement in cognitive abilities, in comparison to those who do not indulge in gaming activities.

## INTRODUCTION

Cognition is the collection of all psychological abilities and methods related to knowledge, attention, working memory, decision, evaluation, problem solving and decision making. Cognitive methods make use of existing knowledge and help generate new ideas. The use of video games in young population has exponentially grown in recent years with claims that such computer based games can enhance cognitive functions of the concerned individual.^1^ The differentiating feature between video games and other media lies in their quality of being interactive. Gamefication’ is increasingly becoming a technique to motivate leaners although very less empirical evidence is documented to support its use.^2^ Video games are considered to be most technologically demanding as well as psychologically absorbing experience for the mental functioning.^3^

Students prefer learning new things in the form of games instead of the orthodox methods of reading books.^4^ Video games have deeply penetrated into the lives of children as well as young adults with studies proving that over 97% of the children in the United States play games for at least one hour per day.^4^,^5^ In USA video games brought in more than $25 billion, which is more than Hollywood’s box office sales in USA and Canada.^6^ Even in Pakistan over 20-million of the population is increasingly getting addicted to internet and is playing the video games both online and offline.^7^

Playing video games may lead to aggression, addiction and depression among the individuals.^8^,^9^ However researchers hove now started to document the benefits of video games in terms of enhancing mental health and well-being in children.^10^,^11^

Due to negative findings related to gaming addiction this topic has been identified by the media mostly leading to public condemnation of playing such games.^12^ Still game playing is believed to promote ICT skills and sports training as well as formal education. Playing video games improve the ability to sustain focus as well as successfully multitask among older adults.^13^

Studies in the West have been carried out to explore the harm associated with gaming, but there is a need to explore positive aspect of playing video games in Pakistani children community. As playing video games is increasing nationally, a proforma-based study among the school going children of Pakistani community is conducted to assess the cognitive abilities of children.

## METHODS

A comparative cross sectional study was conducted at two schools in Rawalpindi from August 2014 till February 2015. Using WHO sample size calculator, 171 sample size was calculated with Confidence Level (CL) of 95%, Anticipated population proportion (P) of 0.5 and Absolute precision (d) of 0.15). Permission from ethical review committee was taken. After informed verbal consent from children parents or guardians, convenient sampling technique was used to induct students into study. Those students were inducted who were between 15 to 21 years of age of both genders. Those students were excluded who were mentally or physically unfit or unwilling to participate. A mixed questionnaire was used as a data collection tool. Two groups were defined as Video gamers and non-video gamers. Video gamers were those students who using video games (2D or 3D, racing, shooting etc) for two hours or more. Student playing video games less than two hours were categorized as non-video gamers.

Cognitive ability of the students were assess by Wonderlic Cognitive Ability Test Questionnaire,^14^ a standardized questionnaire, which has to be answered within 12 minutes time, aiming cognitive functions such as knowledge, analogy, processing speed, deductive reasoning and mathematical intelligence. Their score was graded on a scale of one to four. All numbers from one to four were interpreted accordingly. At the end of the questionnaire, individual scoring was done by simple unitary method, which were graded as follows:

Data were entered and analyzed in STATA version 14. Qualitative data was presented as frequencies and percentages. Quantitative data was presented as mean and standard deviation such as age. Chi square test of significance was used to find association between the groups and qualitative variables such as gender and cognitive abilities. Independent sample t test was applied among group and age. A p-value of < 0.05 was taken as statistically significant.

## RESULTS

To assess the cognitive abilities total 171 participants were included in the study out of which 93 (54.4%) were gamers whereas 78 (45.4%) were non-gamers. Mean age of the participants was 18.86±1.3 years. About 116 (67.8%) were males whereas 55 (32.2%) females participated in the study. Details are shown in Table-II.

**Table-I:** Wonderlic cognitive ability test questionnaire scoring..

Scores	Grading
1	Poor
2	Average
3	Good
4	Best

**Table-II:** Demographic variables of the participants (n=171).

Variables	Gamers (n = 93) n (%)	Non-Gamers (n= 78) n (%)	p-value
*Age in years*			
Mean±SD	18.86±1.3		0.35*
*Gender*			
Males	70 (40.9)	46(26.9)	
Females	23 (13.5)	32(18.7)	0.188**
*Hours spent playing*	-		
Not at all	-	34 (19.9)	
0-1hrs	63 (36.8)	44 (25.7)	
2-3 hrs	23 (13.5)	-	-
4-5 hrs	5 (2.9)	-	
6-7 hrs	2 (1.2)	-	
8 hrs or more		-	
*Types of Games*			
Shooters	20(11.7)		
Sports	46 (26.9)		
Online multiplayer	30 (7.02)		
Action	30 (17.54)		
Stimulation	17(7.02)		
Strategy	17 (9.94)		-
None	34 (19.88)		

* Independent sample t test,

** Chi square test.

Participants not playing games at all were 34 (19.9%), 44 (25.7%) played for less than one hour, those playing for 2-3 hours were 63 (36.8%) whereas those playing for more than 4 hours were 30 (17.54%).

**Fig.1:**
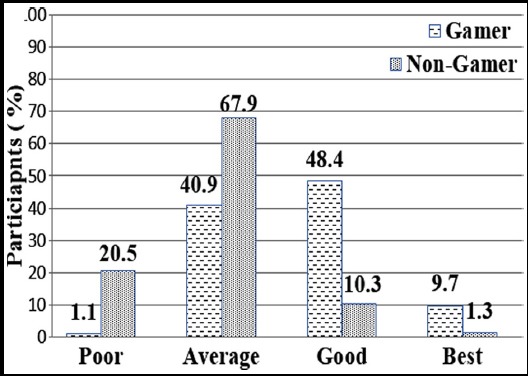
Cognitive ability grades among gamers and non gamers (P<0.001).

During analysis of cognitive ability grades among gamers group, it was found that there were 1 (1.1%), 38 (40.9%), 45 (48.4%) and 9 (9.7%) participants as poor, average, good and best respectively. While among non-gamers, the participants grades were 16 (20.5%), 53 (67.9%), 8 (10.3%) and 1 (1.3%) as poor, average, good and best respectively. There was a statistically significant association among the group and cognitive ability score (p<0.001). Details are shown in Fig.1.

**Fig.2:**
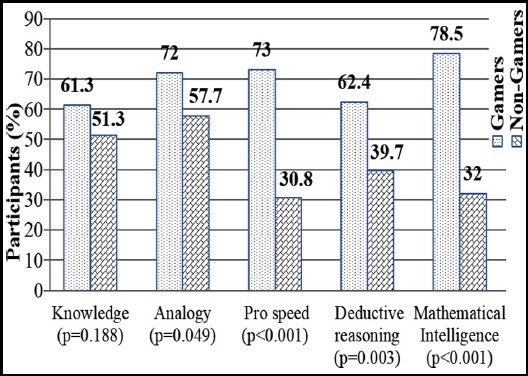
Correct cognitive responses of gamers verus non-gamers.

Gamers with correct answers to ‘knowledge’ segment were 57 (61.3%), while non-gamers who showed up with correct answers were 40 (51.3%). Analogy portrayed 67 (72%) of gamers with correct options, while non-gamers exhibiting correct scores were 45 (57.7%). Sixty-eight (73%) of gamers exhibited good processing speed, while non-gamers with appreciable processing speed were 24 (30.8%). Fifty-eight (62.4%) of gamers turned up with significant ability of deductive reasoning, while non-gamers found to be of comparable standard were only 31(39.7%). Gamers with remarkable mathematical intelligence were 73(78.5%), but on the contrary, non-gamers with perfect scores were 25 (32%). Significant association was found between Gender and Gamer status p=0.023). Knowledge and Gamer status (p=0.188); Analogy and Gamer status (p=0.049); Processing speed and Gamer status (p<0.001); Deductive Reasoning and Gamer status (p=0.003); Mathematical intelligence and Gamer status (p<0.001). Details are shown in Fig.2.

## DISCUSSION

Games challenge our mental skills in a way that performance in other tasks involving the same abilities, improves. In a study held in 2013, a double-blind randomized clinical trial was conducted using a mind training game and a quiz game. A total of 32 individuals were enrolled. Before and after training, cognitive function were recorded. It was concluded that executive roles, work memory and processing speed were all improves in commercial mind training games.^15^ In our study we considered 171 participants who were tested with close ended questionnaires consisting of 13 questions from Wonderlic Cognitive Ability sample test. Time given to complete the test was three minutes and 30 seconds. Both gamers and non-gamers were included. Results were analyzed and comparison was made. Gamers showed overall higher scores.

A study was conducted to find whether training older adults with video games increases their cognitive working and results were compare before and after training intervention. In the meta-analysis, 20 clinical trials during 1986 and 2013 were included which involved 474 trained and 439 controls adults. It was concluded that there were positive impact on cognitive functions among the video games performing groups including reaction time, memory and attention.^15^ Our study also showed that those playing video games had better knowledge, deductive reasoning, analogy, mathematical intelligence and memory.

In another study, it was showed that the video games which focuses frequently on cognitive functions improves the tasks performance. The results of study proposed that more frequent usage of specific cognitive functions during gaming improves general cognitive functions of the individual.^16^

To find neural basis of superior visual and motor skills a study was conducted in 2015 which indicated that long-term video games playing was linked with greater white matter integrity in the motor and greater-tier visual pathways^17^ and another study also found that association among cortical thickness and duration of video games.^18^ These studies were conducted at a very advanced level involving technology which could assess changes at the neural level which frequent playing of video games could induce. These facts very strongly support positive association between playing video games and cognition.

In one study it was shown that video gaming improves children’s problem solving skill, ability to sustain attention, pro-social behavior and certain non-cognitive tasks. There is no proof that video game playing increases hostility in children as purposed by some people.^19^ Similarly to test that whether older adults could improve multitasking ability by playing games as mental functions deteriorate with age, the researchers randomly assigned 46 healthy adult, with range of age group as 60 to 85 years, to two groups one playing 3D games and the other not playing. Those who played the multitasking game significantly improved their multitasking performance index when the training period was finished. These levels they reached were higher than the untrained ones.^20^ in this study sample, individuals from 15 to 21 years of age with mean age of around 18 years were enrolled.

Assessment before exposure and training via specific game wasn’t done rather participants were randomly chosen from the population and they all had variable experience in gaming. We tested participants with time based Wonderlic cognitive ability test consisting of questions which assess mathematical intelligence, processing speed, memory, deductive reasoning and analogy. Analysis of scores obtained by gamers and non-gamers showed a positive association between playing video games and cognitive ability. Gamers did well in all fields as compared to the non-gamers. However, all participants had variable experience in gaming with all playing different types of games and for different lengths of time. So standardization could not be achieved. Moreover experimental trial would have been more reliable for this comparison but due to shortage of resources it wasn’t possible.

## CONCLUSION

Gamers exhibit better range of cognitive abilities specifically involving knowledge, analogy, processing speed, deductive reasoning, and mathematical intelligence. In this study, those who play video games on long term basis, showed improvement in cognitive abilities, in comparison to those who do not indulge in gaming activities suggesting as gaming activities trigger enhancement in brain functions.

## RECOMMENDATIONS

More studies should be planned under controlled conditions using advanced tools for cognitive assessment for a more reliable comparison of gamers and non-gamers. Recruitment centers of school and colleges can use video games for testing and short-listing individuals.
